# The Utility of N-Terminal Pro-Brain Natriuretic Peptide as an Adjunct Diagnostic Tool for Acute Heart Failure in Acute Dyspneic Patients Coming to the Emergency Department: A Retrospective Review of Our Early Experience

**DOI:** 10.21315/mjms2021.28.4.15

**Published:** 2021-08-26

**Authors:** Rathika Rajah, Kuan Yee Lim, Boon Hau Ng, Chun Ian Soo

**Affiliations:** 1 Department of Internal Medicine, Universiti Kebangsaan Malaysia Medical Centre, Kuala Lumpur, Malaysia; 2 Respiratory Unit, Department of Medicine, Universiti Kebangsaan Malaysia Medical Centre, Kuala Lumpur, Malaysia; 3 Division of Respiratory Medicine, Department of Internal Medicine, University of Malaya Medical Centre, Kuala Lumpur, Malaysia

**Keywords:** acute heart failure, N-terminal pro-brain natriuretic peptide

## Abstract

Acute dyspnea is one of the prevalent reasons for admission to the emergency department. The use of N-terminal pro-B-type natriuretic peptide (NT-proBNP) as an adjunct for assessing acute dyspnea is not a common practice in many public hospitals in Malaysia. This retrospective review is part of our clinical audit to determine the utility of NT-proBNP as an adjunct to non-standardised clinical evaluation in identifying acute heart failure (HF) in patients with persistent dyspnea (24 h) post-admission. In this cohort of 30 patients with acute dyspnea, NT-proBNP was positive in 20 patients (87%) with acute HF. Three patients (13%) who were treated for septic shock recorded a NT-proBNP false-positive. NT-proBNP demonstrated an overall sensitivity of 90%, a specificity of 70%, a positive predictive value of 85.7% and a negative predictive value of 77.8% in identifying acute HF. These results reinforce that age-stratified NT-proBNP cut-off values are useful for ruling-in or -out acute HF. Thus, NT-proBNP should be considered a crucial point of care, testing to decifer the conundrum of acute dyspneic patients.

## Introduction

Dyspnea is an extremely common nonspecific complaint in patients presenting to the hospital. At times, it can be challenging for clinicians to formulate an early and accurate diagnosis. B-type natriuretic peptide or its prohormone N-terminal pro–B-type natriuretic peptide (NT-proBNP) are biomarkers that have been extensively utilised to exclude heart failure (HF) (1, 2, 3). These biomarkers also have prognostic values for HF (3). However, these tests are not commonly performed because they are not widely available in many public hospitals throughout Malaysia. This retrospective review aims to determine the utility of NT-proBNP as an adjunct to non-standardised clinical evaluation in identifying acute HF in patients with dyspnea.

## Methods

This retrospective cohort study was conducted in a tertiary hospital in Kuala Lumpur, Malaysia. We included patients with persistent dyspnea (24 h post-admission) referred to the respiratory team between August 2018 and January 2019. We reviewed all patients’ demographics, clinical findings of medical officers (MOs), NT-proBNP and echocardiogram (ECHO) results. Patients with no written consent or without a formal ECHO assessment before discharge were excluded. As this was part of our clinical audit, a sample size calculation was not performed, and the number of patients recruited was based on all who fulfilled the inclusion and exclusion criteria.

Overall, acute dyspneic patients were initially assessed by MOs in the emergency room (ER). The clinical evaluations comprised non-standardise history taking, physical examination, electrocardiogram and a portable chest X-ray (CXR). A bedside ultrasound was also used to approximate the left ventricular ejection fraction (LVEF) through eyeballing. A LVEF below 50% was considered abnormal. Based on the available information, (preliminary) clinical diagnoses were devised to stabilise patients and guide initial treatment. All MOs were postgraduate medical trainees. A registrar or specialist supervised the overall management plans.

Patients with persistent dyspnea were referred and reevaluated by the respiratory subspecialty team. Upon review, all patients were provided an NT-proBNP test (Roche Diagnostics, Germany) with an earlier ECHO (performed by trained cardiac sonographers) assessment. The NT-proBNP results were interpreted based on International Collaborative of NT-proBNP (ICON) study age-stratified cut-off values (rule-in cut off of 450 pg/mL, 900 pg/mL and 1,800 pg/mL for ages < 50, 50 to 70 and > 75 years old, respectively and a rule-out cut off of 300 pg/mL for the diagnosis of acute HF) (2). The diagnosis of HF was based on the European Society of Cardiology (3).

## Statistical Analysis

IBM SPSS Statistics for MAC (version 23.0, Armonk, NY: IBM Corp) was used for all statistical analysis. The mean (standard deviation) and median [interquartile (IQR) range] were used for results of normally distributed grouped data and non-normally distributed data, respectively. A 2 × 2 table was used to analyse the sensitivity, specificity, positive and negative likelihood ratios (LR+/−) with corresponding 95% confidence intervals (CI).

## Results

**Figure 1:**
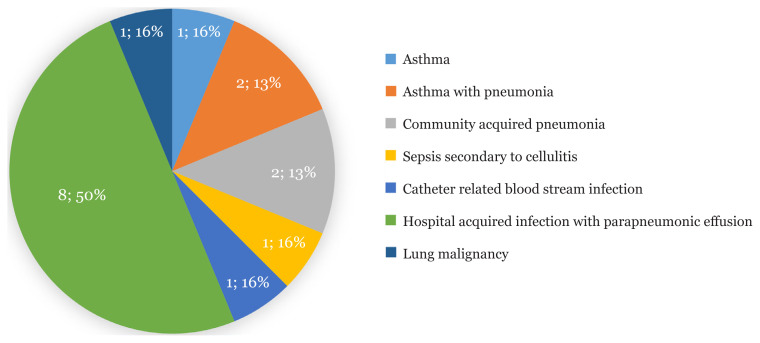
Preliminary diagnoses (misdiagnosed) of cases with acute HF (*n* = 16)

**Figure 2:**
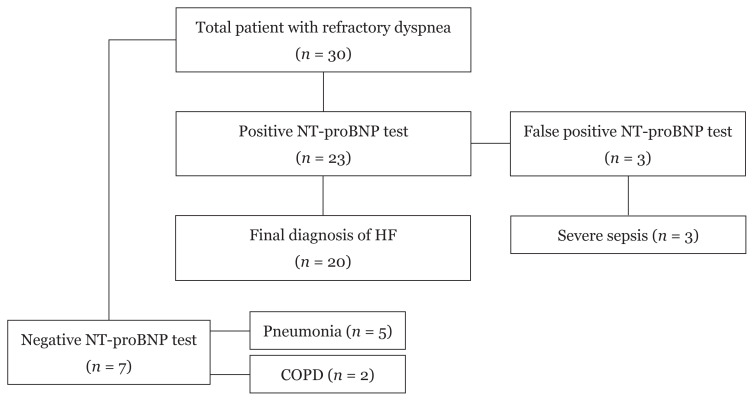
Flowchart demonstrating the outcome of patients referred for persistent dyspnea

Thirty-two patients with various clinical diagnoses for acute dyspnea were referred. Two patients did not have an ECHO done and, thus, were excluded from the study. Twenty-three patients (76.7%) had elevated NT-proBNP levels. After reassessment and availability of ECHO results, 20 patients (67%) had the final diagnosis of acute HF. Sixteen patients (53%) required a revision of their diagnoses due to misdiagnosis of acute HF. Patients with acute HF had a mean age of 65.6 (2.9). Median values of NT-proBNP in non-HF and acute HF were 976.5 pg/mL (IQR 127.25–24,837) and 3510 pg/mL (IQR 1,881–29,292), respectively. The demographics and clinical evaluation of patients with a final diagnosis of acute HF are presented in Table 1. Figure 1 illustrates the preliminary diagnoses provided to cases of misdiagnosed HF in the ER. Three patients (10%) with septic shock had false-positive NT-proBNP levels. They had normal ECHO findings (performed upon resolution of septic shock). The remaining cases of refractory dyspnea (non-HF) were secondary to pneumonia (five patients: 16.7%) and chronic obstructive pulmonary disease (two patients: 6.7%). Figure 2 summarises the outcome of the cases referred.

NT-proBNP demonstrated a sensitivity of 90% (95% CI: 68.3, 98.8), specificity of 70% (95% CI: 34.8, 93.3), positive predictive value of 85.7% (95% CI: 69.7, 94.0) and negative predictive value of 77.8% (95% CI: 46.9, 93.3) in identifying acute HF (Table 2).

## Discussion

Acute dyspnea is a common presenting complaint in the ER. In Malaysia, HF is a leading cause of cardiac-related death (4). A diagnosis of HF commonly relies on the presence of typical signs and symptoms and supporting investigations.

The dependence on conventional history-taking, physical examination, the suboptimal quality of portable chest radiographs and non-standardised bedside cardiac ultrasound techniques may have contributed to more than half of our patients (53%) requiring a revision of their initial diagnoses. Clinical symptoms are often nonspecific in differentiating acute HF from other causes of dyspnea (3, 5). Besides, clinical signs such as elevated jugular venous pressure (JVP), lung crepitations and lower limb oedema are not pathognomonic of HF. Our study demonstrated that only elevated JVP was significantly identified by MOs in patients with HF. Due to its ready availability and low cost, CXR is commonly used to assess dyspnea. It has low diagnostic accuracy in acute HF (6). Some limitations include unfamiliarity with the features of acute HF and suboptimal quality of the portable CXRs. Similarly, ECG has low specificity in the diagnosis and is mainly used to exclude acute HF (3). Due to the high patient load, it is not unusual to encounter a long waiting time to obtain a formal ECHO in numerous Malaysian public hospitals. Therefore, making a correct diagnosis can be challenging for clinicians.

Nevertheless, vigilance in recognising the relevant signs and symptoms and maintaining an open mind when making differential diagnoses are crucial in differentiating the causes of acute dyspnea. A high index of suspicion is needed when dealing with cases of non-typical presentation. When there are limitations in clinical assessment, further ancillary testing can be the key to improving acute HF diagnosis.

NT-proBNP is elevated in the presence of increased stress on the myocardium, presenting as either a volume or pressure overload. The use of NT-proBNP to help distinguish acute HF from other noncardiac causes of acute dyspnea is well described (7, 8). Using age-related cut-off points of 450 pg/mL, 900 pg/mL and 1800 pg/mL for ages < 50, 50–75, and > 75 years old, respectively, a large retrospective cohort study (ICON) that combined and analysed three previous prospective studies demonstrated a 90% sensitivity and 84% specificity for acute HF (1–2). NT-proBNP levels of < 300 pg/mL are a rapid yet accurate method for excluding acute HF with a negative predictive value of 99% (8). Our study’s results also reinforce the use of age-stratified cut-off values whereby NT-proBNP helped rule-in the diagnosis of acute HF. Compared to previous trials, our results showed a similar sensitivity but lower specificity due to false-positive results from patients with septic shock. False-positive NT-proBNP results can be seen in patients with sepsis-related cardiomyopathy (9). NT-proBNP values can also be elevated with chronic kidney disease (CKD) (10). In this study, three patients with CKD had an estimated glomerular filtration rate (GFR) of more than 60 mL/min per 1.73m^2^, and their NT-proBNP levels were significantly above 900 pg/mL (NT-proBNP and GFR cut-off values reported by Anwaruddin et al. (10) for patients with underlying CKD. These results were considered true positives.

Therefore, NT-proBNP can be utilised early as an adjunct to clinical evaluation when the cause of dyspnea is unclear. NT-proBNP can also complement ECHO in HF diagnosis with preserved ejection fraction (HFpEF) and HF with mid-range ejection fraction (HFmrEF) (3). When incorporated with clinical assessment and natriuretic peptide testing, the proposed cardiopulmonary assessment in real-time to determine diastolic and systolic function with sonography (CARDDSS) examination may impact the diagnosis and management of acute HF (11). The CARDDSS approach involves sonographic assessment of pulmonary congestion, central venous filling pressure and LVEF. Likewise, increased exposure to structured training and handling of the ultrasound machine are also essential in order to improve the diagnosis of acute HF.

This review’s limitations include the following factors: single-centre involvement, retrospective review with small sample size and the potential of selection bias, as the patients included were only those being referred.

## Conclusion

NT-proBNP, when used as an adjunct to clinical evaluation, is useful in assisting the diagnosis of acute HF, especially when diagnostic indecision is present.

## Figures and Tables

**Table 1:** Demographics and clinical findings for acute dyspneic patients in the emergency department

Characteristics	HF# (*n* = 20) Frequency (%)	Non-HF# (*n* = 10) Frequency (%)	*P*-value
Age in years*	65.6 (2.9)	62.3 (4.9)	0.099^a^
Gender			
Male	8 (40)	6 (60)	0.442^c^
Female	12 (60)	4 (40)	
Comorbidities			
Hypertension	16 (80)	6 (60)	0.384^c^
Diabetes mellitus	13 (65)	4 (40)	0.255^c^
Chronic kidney disease	3 (15)	1 (10)	1.000^c^
History of cardiac failure	8 (40)	2 (20)	0.419^c^
Physical signs			
Jugular venous pressure			
Elevated	7 (35)	1 (10)	0.027^b^
Normal	4 (20)	7 (70)	
Not assessed	9 (45)	2 (20)	
Bilateral lower limb oedema			
Present	14 (70)	6 (60)	0.690^c^
Absent	6 (30)	4 (40)	
Not assessed	0 (0)	0 (0)	
Lung auscultation			
Crepitations	13 (65)	5 (50)	0.705^b^
Reduced breath sound	6 (30)	4 (40)	
Expiratory rhonchi	1 (5)	1 (10)	
Lung percussion	NP	NP	
Cardiac impulse assessment			
Displace apical impulse	NP	NP	
Third hear sound (gallop rhythm)	NP	NP	
Electrocardiogram			
Sinus rhythm	14 (70)	7 (70)	1.000^c^
Atrial fibrillation or flutter	6 (30)	3 (30)	
Left ventricular hypertrophy	10 (50)	4 (40)	0.709^c^
Left bundle branch block	1 (5)	0 (0)	
Chest X-ray interpretation			
Unilateral consolidation	3 (15)	3 (30)	0.321^b^
Bilateral consolidation	4 (20)	2 (20)	
Unilateral effusion	5 (25)	0 (0)	
Bilateral effusion	2 (10)	0 (0)	
Interstitial abnormalities	4 (20)	2 (20)	
Consolidation with effusion	2 (10)	3 (30)	
Cardiomegaly	4 (20)	1 (10)	0.640^c^
Bedside cardiac ultrasound in the emergency department			
Approximation of abnormal LVEF < 50%	4 (20)	1 (10)	0.640^c^
NT-proBNP (pg/mL)±	3,510 (1,881–29,292)	976.5 (127.25–24,837)	

Notes:

* Values are presented as mean (standard deviation) for continous variables;

± median (IQR) for non-continous variables;

a *P*-values are calculated, where appropriate, using independent *t*-test;

b Chi-square test;

c Fisher’s exact test;

# Final diagnosis of HF (including cases with diagnosis revision);

NP = not performed

**Table 2:** The performance of NT-proBNP in identifying acute HF in patients with persistent dyspnoea (*n* = 30)

NT-proBNP	
Sensitivity (95% CI)	90% (68.3, 98.8)
Specificity (95% CI)	70% (34.8, 93.3)
Positive predictive value (95% CI)	85.7% (69.7, 94.0)
Negative predictive value (95% CI)	77.8% (46.9, 93.3)
